# Impact of surface topography and hydrophobicity in varied precursor concentrations of tenorite (CuO) films: a study of film properties and photocatalytic efficiency

**DOI:** 10.1038/s41598-024-58744-x

**Published:** 2024-04-04

**Authors:** Mohammed Althamthami, Hachemi Ben Temam, Elhachmi Guettaf Temam, Saâd Rahmane, Brahim Gasmi, Gamil Gamal Hasan

**Affiliations:** 1 Physics Laboratory of Thin Films and Applications, Biskra University, BP 145 RP, 07000 Biskra, Algeria; 2 University of El Oued, N48, 30000 El Oued, Algeria

**Keywords:** Surface roughness, Wettability, Tenorite, Photocatalysis, Dip-coating, Pollution remediation, Chemical engineering

## Abstract

Semiconductor films are crucial in photocatalysis applications, yet their controlled production remains challenging. Previous studies have mainly focused on deposition processes, heating rates, and doping of semiconductor oxides. In this paper, we introduce a novel method for fabricating tenorite (CuO) semiconductor films with varying precursor concentrations (0.01, 0.02, 0.04, 0.06, and 0.1 g/ml) using a dip-coating technique. We explore the impact of contact angles, 3D surface topography, and film thickness on photoactivation properties, areas with limited previous research focus. The results demonstrate that higher-concentration tenorite films (0.1 g/ml) exhibit rougher surfaces (77.3 nm), increased hydrophobicity (65.61*°*), improved light-harvesting ability, enhanced charge separation, and higher active oxygen output. The crystal sizes were within the range of 7.3–44.1 nm. Wettability tests show a 21.47% improvement in the 0.1 g/ml film surface under indirect sunlight compared to darkness. Transmittance rates in the 600 nm range were from 0.02 to 90.94%. The direct optical band gaps were 1.21–2.74 eV, while the indirect band gaps remained unaffected (0.9–1.11 eV). Surface morphology analysis reveals an increased presence of grains with higher concentrations. Regarding photocatalysis's impact on film morphology and copper content, SEM images reveal minimal changes in film structure, while copper content remains stable with slight variations. This suggests strong adhesion of tenorite to the film after photocatalysis. Tenorite thin films display exceptional photocatalytic efficiency, making them suitable for practical applications.

## Introduction

Freshwater is among the most fundamental needs of humanity on planet^1^, and it has become a big issue in the twenty-first decade^2–4^. Over 80% of the total ground and surface waters are contaminated^5^, Every day, 7.5 billion people need clean water for a variety of functions^1^, and according to the UNWWD (United Nations World Water Development) report, throughout the world, nearly 750 million individuals lack access to a reliable source of drinking water, and the need for water in the manufacturing sector is expected to increase 400% by 2050^6^. The problem of water contamination has been increasingly significant^7,8^, since the late twentieth century because of fast economic growth^9^. Several research organizations have focused heavily on pollution-related issues^10–12^. These include pollution from organic and inorganic pollutants^6^, which are generally challenging to decompose by nature itself^13^ and have been widely investigated due to their negative impact on human life and health^14^.

In light of this, photocatalysis utilizing semiconductor photocatalysts emerges as the most cost-effective and promising approach for efficiently harnessing recoverable solar energy^3,15,16^ and it has been widely developed in response to the rising weight of the global energy problem and pollution^17,18^. As a result, one of the world’s biggest concerns is the efficient treatment of dirty water using photocatalytic processes^5^, which are regarded as among the most successful methods of resolving environmental issues. On the other hand, photocatalysis represents one of the most sophisticated water treatment techniques available today^6^, due to its ease of use, low cost, lack of pollution, repeatability, renewable technology, reliance on just oxygen in the atmosphere^13,19^, and the potential to turn solar energy into clean H_2_ fuel^20^. As a function, it is used in a variety of industries, including CO_2_ reduction and N_2_ fixation^21^, and the decomposition of organic molecules in wastewater, disinfecting, H_2_ purification, etc.^22,23^; that can produce electron–hole pairs effectively by absorbing incoming light and subsequently initiating surface redox reactions^20,24^. As well acknowledged, advanced oxidative processes (AOPs) are regarded as an excellent choice for degrading organic contaminants from water, owing to the production of extremely reactive oxygen species (ROS) such as hydroxyl radicals (•OH) and superoxide radicals (O_2_^−^)^25^.

Some photocatalysts are restricted in their practical applicability due to rapid charge carrier recombination and inadequate absorption of visible light^26^. Thus, it is crucial to develop the material's band gap to identify the optimal photocatalyst with a suitable band gap^27^. Consequently, researchers were attracted to fabricating thin films as they effectively control the band gaps of the catalyst materials^33^. Thin films are sol–gel applications with several functions^10^ that provide more flexibility in terms of lower sintering temperatures depending on precursors, reduced cost, improved compositional control, increased bioactivity, and improved coating morphological control^28,29^. It may also be readily synthesized utilizing sol–gel spin-coating or dip-coating procedures, which are complex or impossible to do using conventional synthetic methods^30,31^.

The dip-coating method is a technique for depositing thin layers onto a substrate surface^32^. It is the preferred technology due to its high deposition, chemical composition controllability, production capability, and excellent homogeneity over large areas of deposition. Furthermore, controlling the band gap in transition metal oxides is important^33^ and provides advantages in terms of cost^34^. In addition, it is easily applied to diverse geometric forms of substrates and has a high potential for usage in large-scale commercial manufacturing^35^. The thickness of the coating layer is determined by the coating slurry viscosity, as well as the withdrawn speed and dwell duration^36^.

Cupric oxide exists in two stable compounds: Tenorite (CuO), which has band gap values ranging from 1.2 to 1.9 eV as well as being capable of considerable light absorption despite its low photocatalytic efficacy^37^, and cuprite (Cu_2_O), which has band gap values ranging from 2.0 to 2.2 eV^38^. Because of the monoclinic phase of CuO, it is more thermostable than Cu_2_O, which has a cubic phase^39^. On the other hand, CuO is a prevalent semiconducting metal oxide based on its conductivity and the presence of p-type semiconductors owing to copper gaps in the crystalline lattice^40^, which is sensitive to visible light and has strong photocatalytic oxidation activity in eliminating organic contaminants under both artificial and solar light^41^. Tenorite has appealing properties, including strong electrical conductivity, excellent stability, and high catalytic activity. Furthermore, tenorite is a low-cost, plentiful, and non-toxic semiconductor^42^.

Babu et al.^43^ described the function of Co-doped CuO (CCO) thin films with p-to n-type properties as a nonmagnetic semiconductor through a half-metal transition. Using the Hall Effect to calculate p- to n-type conductivity. The lower resistivity and mobility at 8 at.% of CCO thin film were found to be 07.83 × 10^3^ Ω cm and 0.003 cm^2^ V^−1^ s^−1^, respectively. The findings above show that 8 at.% of CCO thin films have the potential for use in spintronic and optoelectronic devices. Y. Chen et al.^44^ used reactive magnetron sputtering to create CuO (1 1 1) preferred-oriented thin films with a nanoscale wheatear array. It has been discovered that altering the length of the nano wheatear may control the band gaps of CuO films from 1.54 to 1.25 eV. The measurements made by C-AFM for a work function, as well as the band gap, match those made by KPFM. Finally, it is proposed that KPFM, as well as C-AFM, can both offer practical approaches for determining the semiconductor's band gap.

Methylene blue is extensively used in chemical indicators, pigments, biological staining, and other applications due to its inexpensive cost, water solubility, and high colorability^45^. MB has an aromatic ring structure that is difficult to decompose in a water sample under natural circumstances^46^. Importantly, the nervous system will be damaged when MB enters the human body^47^. MB in water produces a variety of health concerns, including eye irritation, breathing difficulties, mental disorientation, vomiting, and excessive perspiration^48^. Adsorption^49^, electrochemical oxidation^50^, and photocatalytic^51^ are now the most extensively utilized techniques for removing MB from water. Among them, photocatalytic technology was adopted due to its advantages in photocatalyst recoverability^52^.

The majority of studies published in the literature, including these previous studies, have focused on semiconductor oxides, the deposition process, doping, heating rate, or co-doping with other materials^53^. The effects of contact angles, 3D surface topography, and film thickness on the photoactivation properties of semiconductor oxide films are relatively unknown. The effects of contact angles and 3D surface topography on the photocatalysis of pure tenorite films under sunlight have been thoroughly investigated for the first time in this study. This approach seeks to generate new concepts for future study and implement tenorite photocatalytic films by investigating the mechanism of tenorite photocatalysis, associated production methodologies (sol–gel by dip-coating method), and varying the manufacturing precursor concentrations (0.01, 0.02, 0.04, 0.06, and 0.1 g/ml) to improve its photocatalytic performance. The following tests were utilized in this paper: UV–visible was applied to analyze the optical properties. The structural properties were studied using XRD. For morphological features, 3D surface topography, water droplet contact angles, SEM, and EDX were utilized. As a dye, MB was utilized.

## Results and discussion

### X-ray diffraction (XRD)

**Figure 1:**
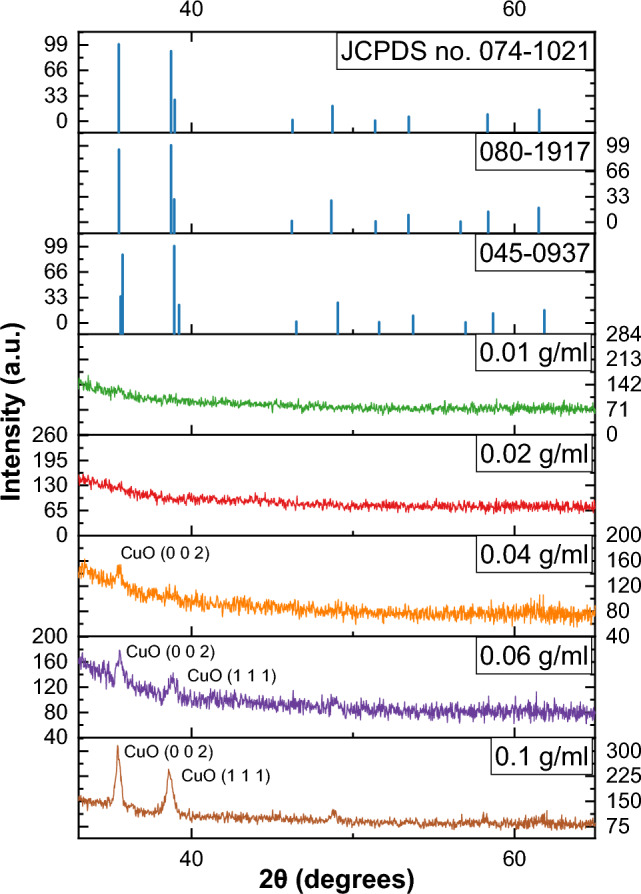
Displays the tenorite thin films XRD patterns for precursor concentrations of 0.01, 0.02, 0.04, 0.06, and 0.1 g/ml.

The findings indicated that films containing precursor concentrations of 0.04, 0.06, and 0.1 g/ml displayed a crystal structure that aligned with the CuO monoclinic crystal system, precisely corresponding to (JCPDS no. 01-074-1021, 080-1917, and 045-0937), while the 0.01 and 0.02 g/ml films exhibited a lower crystallite, as shown in Fig. 1 and Table 1. The size of the crystallites exhibited an upward trend with increasing tenorite precursor concentrations within the films. This is evident from the larger crystallite sizes observed in the 0.1 g/ml film (44.1 nm) compared to the 0.06 g/ml (18 nm) and 0.04 g/ml (7.3 nm) films, as outlined in Table 1. Furthermore, it was observed that augmenting the thickness of the films exerted a discernible impact on crystal growth. This is substantiated by the presence of larger crystal sizes within the 0.1 g/ml film, which also possessed the greatest thickness, measuring at 1276 nm. Overall, these results suggest that controlling the precursor concentrations and thickness of tenorite thin films can be an effective way to control their crystal structure and size, as shown in Table 1.

**Table 1:** Results report.

Parameters	Units	Concentration (g/ml)						
		0.01	0.02	0.04	0.06		0.1	
Crystallographic phase	–	–	–	CuO	CuO		CuO	
Positions	°2Th	–	–	35.501	35.585	38.807	34.45	38.61
Crystal size	nm	–	–	7.3 ± 0.004	18.2 ± 0.071	18 ± 0.08	27.4 ± 0.096	44.1 ± 0.067
Surface roughness (R_q_)	nm	1.41	6.07	12.3	19.4		77.3	
Particles size	nm	13 ± 4	17 ± 6	24 ± 7	36 ± 5		72 ± 8	
Film thickness	nm	28.63 ± 7	73.23 ± 33	152.66 ± 13	266.92 ± 42		1276.85 ± 53.6	
Transmittance	%	~ 90.94	~ 86.38	~ 48.16	~ 30.07		~ 0.02	
Optical band gap (Indirect)	eV	1.05	1.10	0.90	1.09		1.11	
Optical band gap (Direct)	eV	2.74	2.70	1.93	1.71		1.21	
Photocatalytic Efficiency	%	~ 40.08	~ 51.48	~ 63.90	~ 74.42		~ 82.24	
Average of water droplet’s contact	°	23.79 ± 3	36.16 ± 4	43.5 ± 4.5	49.38 ± 7		65.61 ± 6	
Regression coefficient (R^2^)	–	0.96648	0.99401	0.99664	0.9853		0.993	
Rate constant photocatalytic (K_app_)	min^-1^	0,002,704	0,004,028	0,005,742	0,007,320		0,009,365	
Half-time photocatalytic (t_1/2_)	min	256.34	172.08	120.71	94.69		74.01	

### Analysis of contact angles and film thickness

**Figure 2:**
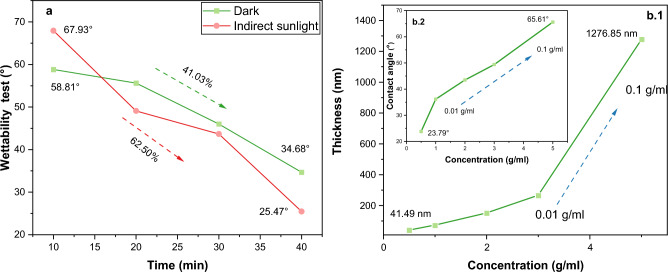
(**a**) Wettability rate of tenorite film surface (0.1 g/ml) over 40 min; (**b.1**) the effect of increasing the precursor concentrations on the thickness of the tenorite films; and (**b.2**) the contact angles.

The contact angle measurements showed that the surface area of the tenorite films (at 0.01, 0.02, 0.04, 0.06, and 0.1 g/ml) had a significant effect on the wettability (hydrophilic), with a maximum value of 65.61° at 0.1 g/ml. The surface energies (γ) of all the film areas were quite high, as all films were less than 90°^54^. Figure 2b.2 shows the average contact angle (water droplet) on the film surface, where the tenorite films were found to be hydrophobic with increasing concentration increased (from 23.79 to 65.61° at 0.01 to 0.1 g/ml). Consequently, this phenomenon results in an augmented self-cleaning effect with the increased precursor concentrations. The higher contact angle value (65.61°) on the tenorite film (0.1 g/ml) surface may be explained by the fact that the nanostructure applied to the surfaces used in this investigation may have improved surface wettability (hydrophilicity) because of the rougher surface (77.3 nm)^55^.

**Figure 3:**
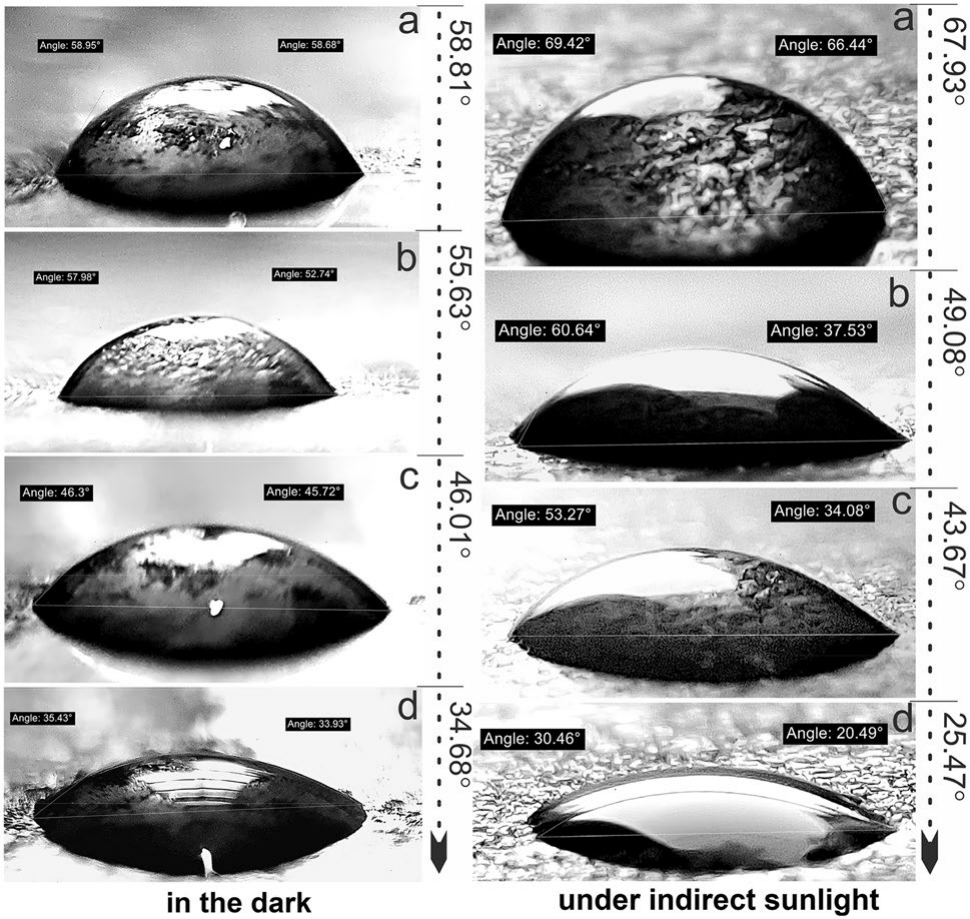
Hydrophilic effect on tenorite film (0.1 g/ml) surface under indirect sunlight and in the dark.

When the tenorite film (0.1 g/ml) was exposed to indirect sunlight for 40 min, the angle of water contact gradually decreased from 69.93° to 25.47°, indicating a hydrophilicity rate of 62.5%, as shown in Fig. 2a. In contrast, in the dark, the contact angle decreased from 58.81° to 34.68° at a rate of 41.03%; this proves that the wettability on the surface of the 0.1 g/ml film increased by 21.47% under indirect sunlight. It was observed that by increasing the illumination period from 20 to 30 min under indirect sunlight, the contact angle remained stable in the 40–50° range. Similarly, after 10–20 min in the dark, the contact angle remained steady in the 55–60° range (Fig. 3); this explains that the wettability is minimal at the beginning of time and increases dramatically after some time (for tenorite films). The contact angle of water on tenorite film (0.1 g/ml) that was maintained in the dark did not shift significantly (with a rate of 41.03%) over the period of 40 min. This results in tenorite films exhibiting hydrophilic behavior when exposed to sunlight. With increasing precursor concentrations, the thickness of the tenorite film significantly increased from 28.63 to 1276.85 nm, as depicted in Fig. 2b.1. This change can be attributed to alterations in the physical and chemical properties of the solution, including viscosity (with increasing precursor concentrations).

### Study of 3D surface topography

**Figure 4:**
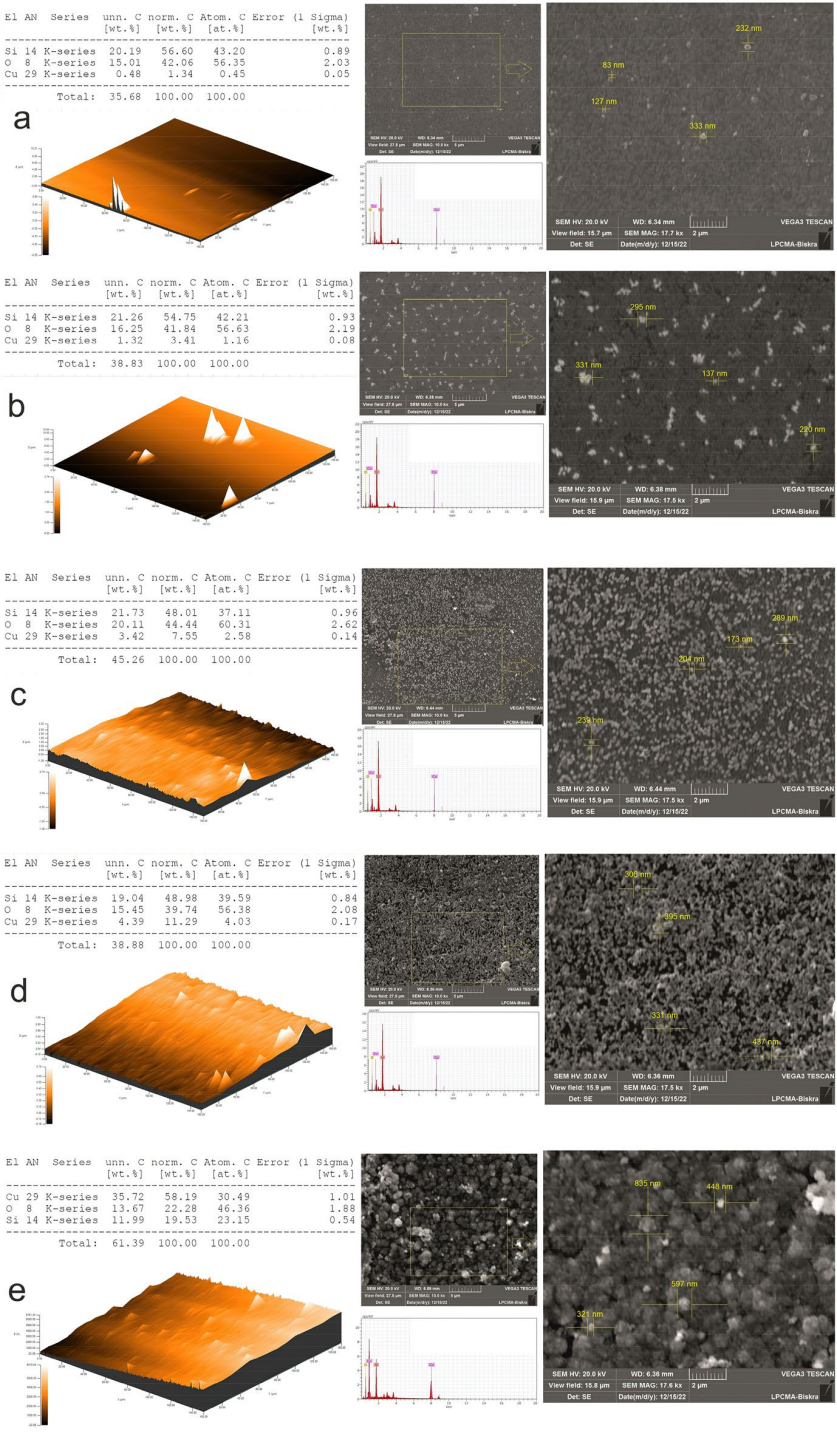
Tenorite thin films 3D surface topography (measurement area: 150 × 150 um) and SEM images with EDX analysis at precursor concentrations of (**a**) 0.01 g/ml; (**b**) 0.02 g/ml; (**c**) 0.04 g/ml; (**d**) 0.06 g/ml; and (**e**) 0.1 g/ml.

The 3D surface topography depicts the surface roughness gradients for the tenorite thin films shown in Fig. 4, which concludes in the results that the surface becomes rougher and more heterogeneous as the precursor concentrations of tenorite increase (from 0.01 to 0.1 g/ml). Root-mean-square roughness (R_q_) values for all films were in the 1.41–77.3 nm range, as indicated in Table 1. A surface roughness value of 77.3 nm was found in tenorite thin film at 0.1 g/ml, increasing the specific surface area of the film (the film is not continuous and has a high level of surface roughness), which makes the photocatalytic activity substantial^10^. The maximum peak heights (R_p_) of the tenorite thin film surfaces were 2.79, 64.3, 60.4, 144, and 299 nm at 0.01, 0.02, 0.04, 0.06, and 0.1 g/ml, respectively. The study encompassed the determination and calculation of various parameters of surface roughness. These parameters, comprising the arithmetic mean roughness (R_a_), quantifying the average deviation of surface height from the mean; skewness (R_sk_), measuring the asymmetry of the surface profile; kurtosis (R_ku_), characterizing the sharpness or flatness of the surface peaks and valleys; total height of the profile (R_t_), representing the overall height variation of the surface; mean height of the profile elements (R_c_), providing the average height of individual surface features; and maximum profile valley depth (R_v_), indicating the deepest valley present on the surface, were determined and are showcased in Table 2.

**Table 2:** Roughness variables' impact on raising precursor concentrations.

Roughness profile parameters	Concentration					Unit
	0.01	0.02	0.04	0.06	0.1	g/ml
*R*_*a*_	1.11	3.13	9.26	13.6	58.9	nm
*R*_*sk*_	- 0.617	5.49	1.08	2.09	0.387	nm
*R*_*ku*_	3.40	49.8	5.56	15.7	3.74	nm
*R*_*t*_	7.40	376.6	91.1	198	523	nm
*R*_*c*_	3.48	10.6	30.5	44.1	170	nm
*R*_*v*_	4.61	12.3	30.7	54.1	223	nm

The roughness parameters (R_a_, R_c_, R_t_, and R_v_) of the 0.1 g/ml film were higher in comparison to the values found in the 0.01, 0.02, 0.04, and 0.06 g/ml films; this explains the dramatic change in the surface (homogeneity and roughness) and morphological structure (Fig. 4) of the films as the precursor concentrations increase. These findings may be described by assuming that increasing the precursor concentrations of the tenorite on the surface of the films causes instability with non-static impacts, resulting in narrower and larger aggregations in the particles of the film’s surface.

### Tenorite thin films EDX analysis patterns and morphological

EDX measurement was utilized to look at the tenorite film's chemical components, which showed the peaks connected to the elements Cu, Si, and O (Fig. 4). SEM images of tenorite thin films unveiled bumpy particle accumulation, suggesting a heterogeneous surface, which intensified with increasing tenorite precursor concentrations ranging from 0.01 to 0.1 g/mL, as depicted in Fig. 4a–e. These particles' expansion can be attributed to rapid diffusion and reduced activation energies when the tenorite concentration increases^56^. Furthermore, the emergence of grains on the films surface increased, with the size of these grains expanding proportionally to the precursor concentrations, resulting in a rougher surface, as corroborated by the roughness results presented in Tables 1 and 2. Moreover, as tenorite precursor concentrations rise (from 0.01 to 0.1 g/ml), average particle size grows from 13 ± 4 to 72 ± 8 nm. The presence of a heterogeneous surface increases the amount of MB adsorbed on these surfaces, enhancing the photocatalytic efficiency of the films^10^.

### UV–visible spectral analysis

**Figure 5:**
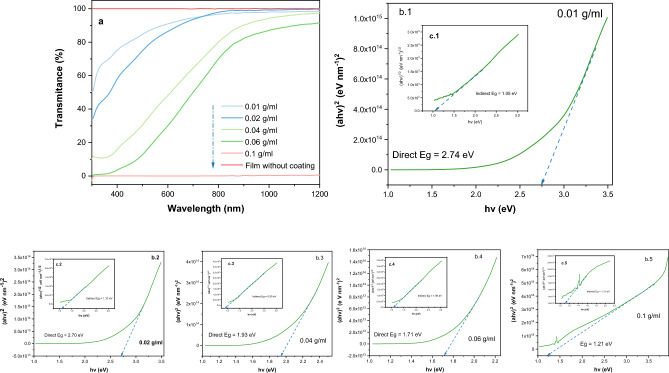
(**a**) visible light spectrum of tenorite thin films after seven dips as a response to the precursor concentrations (0.01, 0.02, 0.04, 0.06, and 0.1 g/ml); (**b1**–**b5**)) representation of the Tauc and Menth plot's for the direct optical band gap (Eg); and (**c1**–**c5**)) indirect band gap for tenorite films.

Tenorite thin films transmission spectra show extremely low to high transparency in the visible region. Figure 5a represents a light spectrum with a variety of outcomes from tenorite films, with transmittance rates ranging from 0.02 to 90.94% at the wavelength (600 nm). The results of 0.01, 0.06, and 0.1 g/ml films show the effect of the amount of oxygen (42.06, 39.74, and 22.28 C norm. [wt%]) and film thickness (28.63, 266.92, and 1276.85 nm) on the transmittance (0.02, 30.07, and 90.94%), respectively. The optical transmittance decreases dramatically (to the point of impermeability as 0.1 g/ml film) as the film thickness of the tenorite increases. The variation of the optical band gap for tenorite thin film was shown by the Tauc and Menth plot in Fig. 5b1–b5 for direct and Fig. 5c1–c5 for indirect. The direct and indirect band gap (transitions) were simultaneously observed within the same films, an occurrence attributed to the combined influence of the films' monoclinic crystal structure, variable particle size, and crystallite dimensions. Within this context, the films exhibited a spectrum of crystal size variations, spanning from a lower crystallite structure (0.01 and 0.02 g/ml) to nanometer-scale dimensions (7.3, 18.2, and 27.4 nm) at precursor concentrations of 0.04, 0.06, and 0.1 g/ml, respectively. These distinctions in crystal size intricately corresponded to discernible alterations in the band gap characteristics. In particular, the direct band gap, extending from 1.21 to 2.74 eV, exhibited a clear association with crystal size, revealing a narrower energy band gap as crystal size increased from 7.3 to 27.4 nm. Conversely, crystal size variations did not exert a significant impact on the values of the indirect band gap, which ranged from 0.90 eV to 1.11 eV. The direct band gap values of tenorite films (ranging from 0.01 to 0.1 g/ml) showed a significant decrease from 2.74 to 1.21 eV when the particle size increased from 13 to 72 nm. In contrast, there was no discernible change in the indirect bandgap values in response to changes in particle size (as detailed in Table 1). Tripathi et al. engineered the CuO’s band gap (a p-type semiconductor) from an initial indirect band gap of ∼1 eV to a direct band gap of ∼4 eV, achieved through precise adjustments in nanostructure morphology and mid gap defect states^57^. As shown in Table 1, the indirect band gap values (1.05–1.11 eV) of tenorite films were not affected by changing the precursor concentrations of Cu(NO_3_)_2_· xH_2_O, the amount of Cu, the crystal size, or the film thickness. Conversely, increasing these factors led to lower direct band gap values (from 2.74 to 1.21 eV) for tenorite thin films. This suggests that the precursor concentrations of CuO in the films can influence their electronic properties. This influence is probably due to changes in film structure, composition^58^, or reduction in oxygen content, which is a consequence of the increased film density enabled by the increased film thickness.

### Photodegradation of MB dye under sunlight irradiation

**Figure 6:**
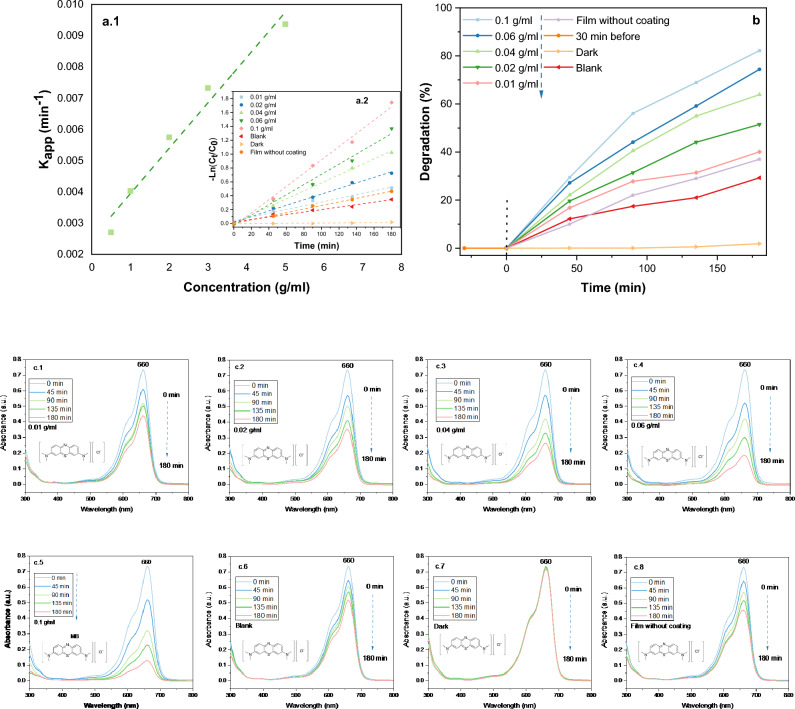
(**a.1**) k_app_ values (constant rate) per precursor concentrations (0.01, 0.02, 0.04, 0.06, and 0.1 g/ml); (**a.2**) Final MB concentration concerning first MB concentration (–Ln(C_t_/C_0_)) for 180 min; (**b**) degradation rate of all tenorite films in relative to 180 min (with tests of dark, blank, and 30 min before); (**c1**–**c8**)) The absorption of MB about 180 min for tenorite thin films with dark and blank.

CuO films show a pronounced upward trend in photocatalytic performance when the precursor concentrations of CuO thin films increases from 0.01 to 0.1 g/ml, leading to a significant improvement in photocatalytic efficiency from 40 to 82%. This phenomenon can be attributed to the heightened density of active sites or enhanced light absorption achieved at elevated concentrations^59^. Figure 6c1–c8 shows graphs depicting the degradation of MB throughout the irradiation period (180 min) of tenorite thin films, as well as graphs depicting the deterioration of MB solution in sunlight without a film and in a darkened room. In consideration of the relationship between hydrophilicity and photocatalysis, it was observed that a higher contact angle of water droplets on the film surface correlates with increased efficiency of photocatalysis (Table 1). However, it is important to note that this does not necessarily imply a direct causation where greater hydrophobicity leads to enhanced photocatalysis. This observation arises from tests conducted by fully immersing the film in the MB solution. Nevertheless, such properties could offer significant benefits for self-cleaning water applications^60^. Regarding the influence of surface roughness on photocatalyst efficiency, findings indicate that an increase in roughness, ranging from 1.41 to 77 nm, corresponds to an enhancement in photocatalytic efficiency. This enhancement is observed to escalate from 40 to 82% for CuO films with precursor concentrations spanning from 0.01 to 0.1 g/ml, respectively. Figure 6a.2 depicts the progression of the MB ratio oxidation process −Ln(C_t_/C_0_) throughout degradation time. The film with a concentration of 0.1 g/ml exhibited the highest photocatalytic efficiency, as depicted in Fig. 6b. This phenomenon can be attributed to both the rough surface morphology (Fig. 4) and the highly crystalline state of the film (Table 1). Furthermore, EDX analysis indicates that increasing the amount of the Cu component influences photocatalytic efficiency (Table 1).

The wind velocity, amount of solar radiation, humidity^61^, and temperature affect the photocatalytic process. Table 3 demonstrates the difference between these parameters for each 45 min from 10_a.m_ to 1_p.m_. In Fig. 6b, 0.06 and 0.1 g/ml films with a high thickness at (266–1276 nm) demonstrated a high photocatalysis rate of (74–82%), respectively. This study compares its results with previous research on photocatalysis, focusing on the films produced and the conditions involved, as outlined in Table 4.

**Table 3:** Changes in wind, temperature, humidity, and sun radiation every 45 min at the MB breakdown test on October 15, 2022 (Biskra, Algeria).

Time (min)	0	45	90	135	180
Wind speed (km/h)	16	15	15	12.5	9.2
Day temp (C^0^)	28.5	29.2	29.7	30.7	31.1
Humidity (%)	38.6	37.4	36.7	34.3	33.2
Radiation amount	Moderate	High			Moderate

**Table 4:** Presents a comparative analysis between the findings of this study and those of prior research endeavors pertaining to photocatalysis.

Film materials	Dye type and concentration	Technique type	Degradation (%)	Time (min)	Irradiation Source	References
CuO (0.1 g/ml)	Methylene blue, 0.0022 g/l	Dip-coating (glass Substrate)	82	180	Visible light	Current study
CuO NWs	Methylene blue, 0.0012 g/l	Porous CuO nanowire (NW) matrix	34	180	Visible light	^62^
CuO@TiO_2_-300	Methylene blue, 0.0012 g/l	ALD process was performed on CuO NW	42	180	Visible light	^62^
CuO/ZnO (Simple A)	Methylene blue, 0.005 g/l	Spin-coating with Glass Substrate	44	120	Xe lamp of 150 W	^63^
Cu_2_O	Methylene blue, 0.005 g/l	Spray pyrolysis growth technique	60	120	solar simulator (Dayan) 100 mW/cm^2^	^64^
CuO	Methylene blue, 0.003 g/l	Spraying (glass Substrate)	74	360	Visible light	^65^
CuO-ZnO (1:3)	Methylene blue, 0.003 g/l	Spraying (glass Substrate)	85	360	Visible light	^65^
Cu:Co (70:30)	Methylene blue, 0.003 g/l	Dip-coating (glass Substrate)	78	240	Visible light	^7^
CuO	Rhodamine-b, 0.01 g/l	Sputtering and thermal annealing (glass Substrate)	56	120	250W Xenon lamp	^66^

**Figure 7:**
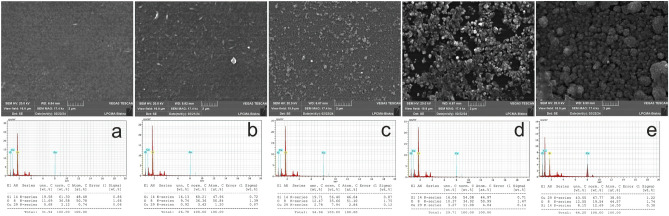
Showcases SEM and EDX images following the photodegradation of MB at the various precursor concentrations: (**a**) 0.01 g/ml, (**b**) 0.02 g/ml, (**c**) 0.04 g/ml, (**d**) 0.06 g/ml, and (**e**) 0.1 g/ml.

The impact of direct band gaps on photocatalysis was observed to demonstrate that as the values of direct band gaps decrease (from 2.74 to 1.21 eV), there is a corresponding increase in photocatalytic efficiency (from 40.08 to 82.24%). In contrast, concerning indirect band gaps, no discernible relationship was identified to the enhancement or reduction of photocatalytic efficiency. Regarding the impact of photocatalysis on film morphology and copper content, SEM images in Fig. 7 reveal minimal differences in film morphology. Additionally, copper content remains relatively unchanged, with slight variations observed (Fig. 7). This suggests strong adhesion of tenorite to the film post-photocatalytic.

**Figure 8:**
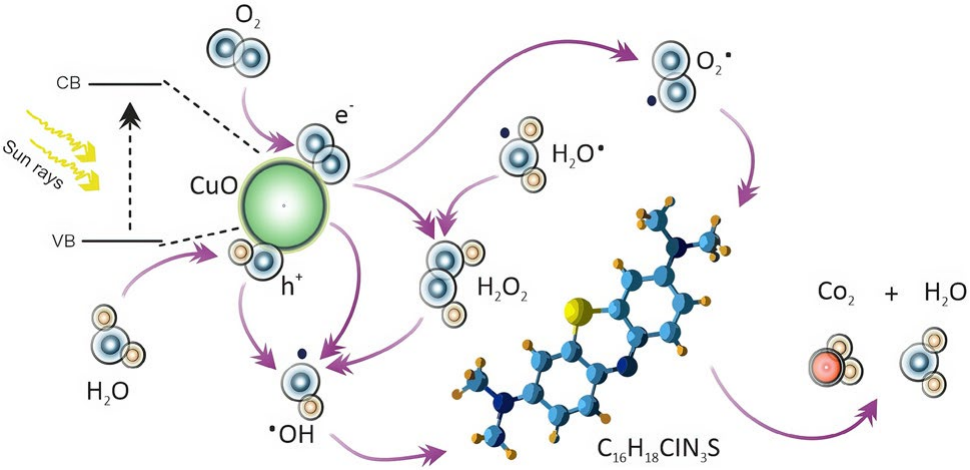
The photocatalytic mechanism of tenorite in MB under sunlight.

The photocatalytic mechanism is illustrated in Fig. 8, while the ensuing equations succinctly encapsulate the photocatalytic properties exhibited by tenorite films in methylene blue solution^37^:

1 $$CuO\xrightarrow {{hv (\left( {\text{solar irradiation}} \right)}} CuO \left( {{\raise0.7ex\hbox{${e^{ - } }$} \!\mathord{\left/ {\vphantom {{e^{ - } } {h^{ + } }}}\right.\kern-0pt} \!\lower0.7ex\hbox{${h^{ + } }$}}} \right) \to CuO\left( {e_{cb}^{ - } } \right) + CuO\left( {h_{vb}^{ + } } \right)$$

Following that, the generation of ${{O}_{2}}^{\cdot -}$ is stated as follow:

2 $$CuO\left( {e_{cb}^{ - } } \right) + O_{2} \to O_{2}^{ \cdot - }$$

Hydroxyl radicals carry free electrons, while superoxide anion radicals have an extra negative charge. Considering their chemical properties, it's sensible to start the degradation process with hydroxylated radicals that have few ions in solution. This sets the stage for the tenorite photocatalysis process^37^:

3 $$CuO\left( {h_{vb}^{ + } } \right) + H_{2} O \to OH^{ - } + H^{ + }$$

4 $$HO_{2}^{ \cdot } + HO_{2}^{ \cdot } \to O_{2} + H_{2} O_{2}$$

5 $$CuO\left( {e_{cb}^{ - } } \right) + H_{2} O_{2} \to OH^{ \cdot } + OH^{ \cdot }$$

6 $$CuO\left( {h_{vb}^{ + } } \right) + H_{2} O/OH^{ - } \to OH^{ \cdot }$$

Moreover, due to anions' natural charge, their interactions should cause significant mineralization with minimal hydroxylated radicals and a realistic level of inorganic ions, if present in the organic structure. After photocatalysis produces superoxide anions ($O_{2}^{ \cdot }$)) and hydroxyl radicals (${OH}^{\cdot }$), they react with organic molecules (MB) as detailed^37^:

7 $$(O_{2}^{ \cdot - } + OH^{ \cdot } ) + MB \to H_{2} O + CO_{2}$$

The photo-electrons convert dissolved oxygen into $O_{2}^{ \cdot }$ radicals that decompose MB. At the same time, the gaps in the valence band (vb) oxidize water via the $OH^{ \cdot }$ radical, extending carrier lifetime and preventing recombination.

The first-order concept is extensively used to describe the kinetics of MB photodegradation (Eq. 15), which allows the integration:

8 $$ln\left( {C_{0} /C_{t} } \right) = K_{app} .t$$

The −Ln(C_t_/C_0_) progressions are linear for each initial concentration, indicating unstable K_app_ values (0.002704 to 0.009365 min^-1^) with increasing precursor concentrations on tenorite films (0.01 to 0.1 g/ml) as shown in Fig. 6(a.1). R^2^ (the regression coefficient) was ~ 0.99 (a stable value according to the Langmuir–Hinshelwood hypothesis). It is possible to determine the half-time (t_1/2_) using the K_app_ parameters:

9 $$t_{{{\raise0.7ex\hbox{$1$} \!\mathord{\left/ {\vphantom {1 2}}\right.\kern-0pt} \!\lower0.7ex\hbox{$2$}}}} = \left( {ln2/k_{app} } \right)$$

**Figure 9:**
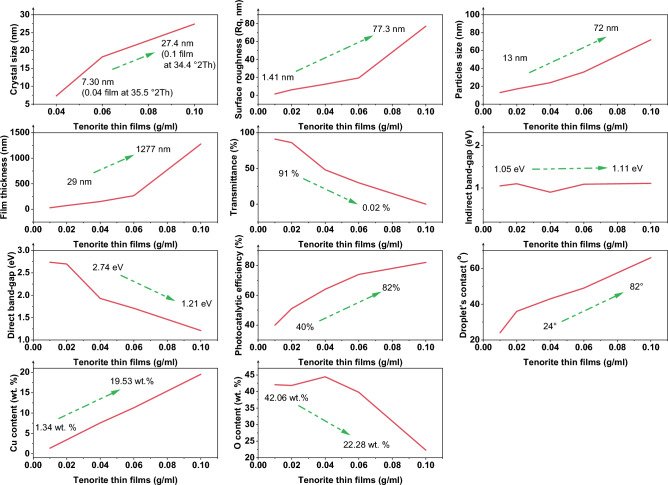
Summary of tenorite thin films properties and their results in this study.

The initial degradation rate increases with concentration (C_0_), driven by t_1/2_ and K_app_ values. Enhanced interaction between MB and photogenerated molecules on tenorite film boosts overall photocatalytic efficiency. Detailed analysis of tenorite thin film properties and their influence on study results, illustrated in Fig. 9.

## Methods

### Materials

Copper(II) nitrate hydrate (Cu(NO_3_)_2_·xH_2_O) (Sigma Aldrich, Product No.: 923087, 99.99%) served as a crucial component in the synthesis of tenorite, the precursor material. To ensure optimal performance, a number of carefully selected solvents and chelating agents, namely ethylene glycol (C_2_H_6_O_2_) (Sigma Aldrich, Product No.: 721972, 99.9 atom % D), 1-Propanol (C_3_H_8_O) (Sigma Aldrich, Product No.: 34871, ≥ 99.9%), and hydrochloric acid (HCl) (Sigma Aldrich Product No.: 320331) were employed. In addition, double-distilled water and hydrochloric acid were used as effective detergents. Finally, methylene blue (C_16_H_18_ClN_3_S) (Sigma Aldrich Product No.: PHR3838), was used for photocatalytic degradation.

### Dip-coating technique of preparing tenorite films

**Figure 10:**
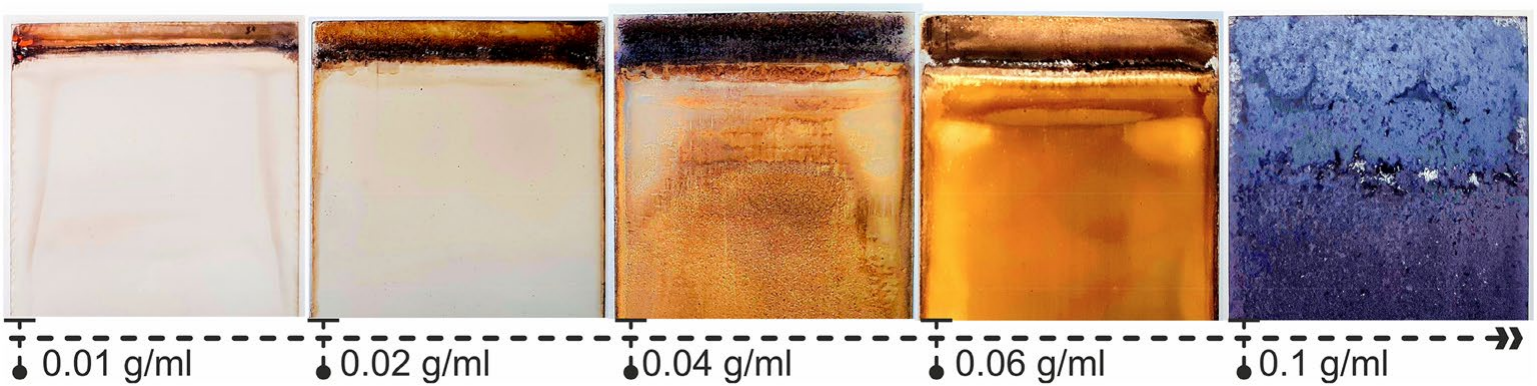
The impact of precursor concentrations (0.01, 0.02, 0.04, 0.06, and 0.1 g/ml) on the morphological structure of tenorite thin films.

To synthesize tenorite films, dissolve 0.5, 1, 2, 3, and 5 g of copper(II) nitrate hydrate in 50 ml of 1-propanol and stir for 30 min until all particles of salt were dispersed (each concentration was prepared individually). Then, for 10 min, 0.5 mL of ethylene glycol and 0.2 mL of hydrochloric acid were added dropwise to the stirring solution to increase viscosity and efficiently disperse throughout the solutions. Finally, continuous stirring was performed until the solution was homogenous (for 1 h). Glass substrates (25.4*76.2*1 mm) in size were immersed in hydrochloric acid and distilled water (20:100%) for 30 min to remove impurities before being washed with double distilled water (DW) and dried with lightly cleaned paper. The films were immersed for 2 min in the solutions with different precursor concentrations of 0.01, 0.02, 0.04, 0.06, and 0.1 g/ml using the HOLMARC apparatus (No. HO-TH-02B). This approach was performed seven times at a withdrawal speed of 9 mm/s. Then, all films were annealed for 30 min at 550 °C with a heating rate of 5 °C/min in an oven (Wisd, Daihan Scientific). The results show that the surface structure of the tenorite films changes significantly with increasing precursor concentrations, as shown in Fig. 10.

### Tenorite films characterization

The structural analysis of tenorite thin films was thoroughly explored through multiple techniques. X-ray diffraction was performed using a state-of-the-art Bruker (D8 Advance model) diffractometer with CuKa radiation (= 1.5406) and a scan range from 10 to 80° at a rate of 0.03° s^-1^ at room temperature. The size of the tenorite crystallites was determined by employing the standard Scherrer's equation on the X-ray diffraction pattern. This technique proved to be highly effective in providing detailed insights into the structural properties of the tenorite thin films.

10 $$D = N\lambda /\left( {\beta {\text{ cos}}\,\theta } \right)$$

where $D$ is the crystallite size, $N$ is a numerical factor, $\lambda$ is the wavelength of X-ray, $\beta$ is half-width maximum, and $\theta$ is the diffraction angle^67^.

**Figure 11:**
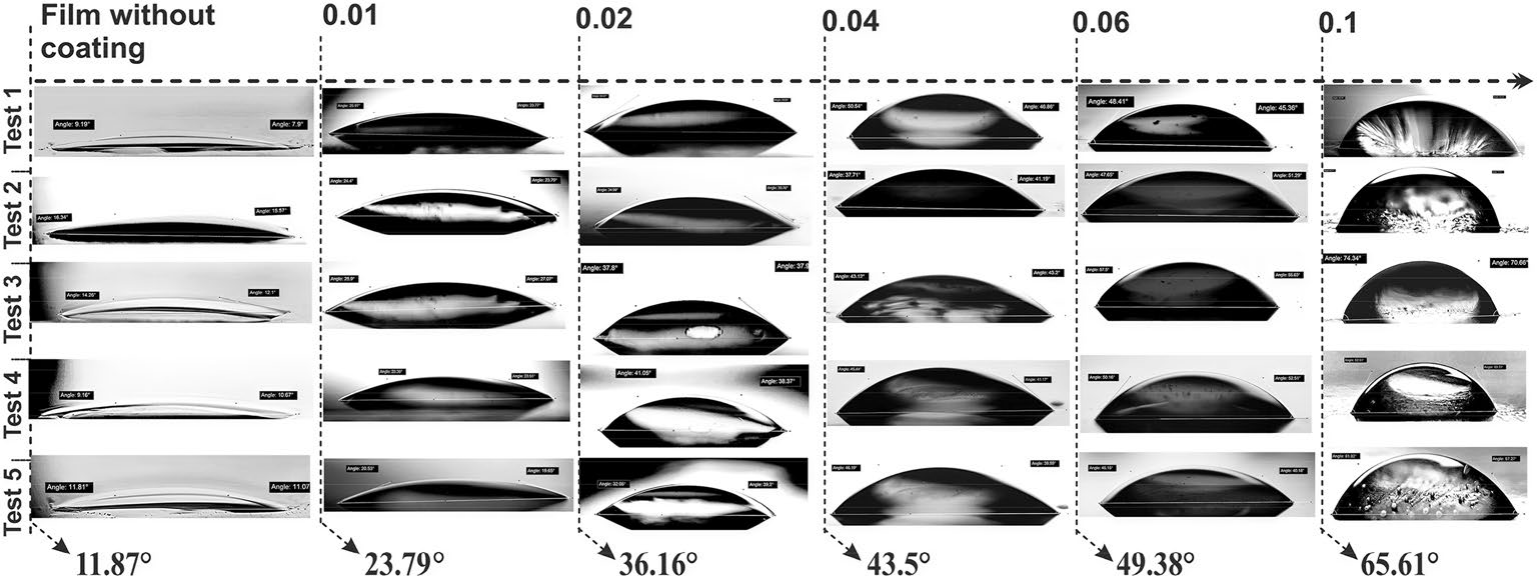
Illustration of tests for calculating the contact angle of a water droplet.

To investigate the hydrophobicity/hydrophilicity of the film surfaces, a handmade approach was employed at ambient temperature. The progression of contact angles was captured using a charge-coupled camera (CCD) connected to an image analyzer. An average value of the water droplet contact angle for each film surface was calculated using the software IC. The contact angles were measured five times carefully using a micropipette (SCILOGEX-iso 9001/13485), corresponding to 10 μl. The average droplet contact angle was calculated using the following equation, as shown in Fig. 11:

11 $$\theta = (\theta_{1} + \theta_{2} )/{ }2$$

where $\theta$ is the ${\theta }_{1}$ and ${\theta }_{2}$ average value (°), ${\theta }_{1}$ is the left angle of the water drop (°), and ${\theta }_{2}$ is the right angle (°).

The film thickness (t) was calculated using the following equation:

12 $$t = m/g.A$$

where *t* is the film thickness (cm), *A* is the film surface area (cm^2^),* m* is the mass of the film (g), and *g* is the film density (g.cm^-3^)^68^. Additionally, the film thickness was calculated for each side of the film (front, back, and bottom, along with the small sides).

A mechanical profilometer (Tencor P-7) was used to measure the 3D surface topography and surface roughness (done in ambient atmosphere and at room temperature) using the 2-bar technique with filter modification (Gaussian filter, 0.800 m cut-off with edge effects).

A scanning electron microscope (SEM, JEOL-JSM 5800) with energy-dispersive X-ray spectroscopy (EDX) was used to investigate the basic chemical composition and morphology.

A UV–vis instrument (JASCO V-770) with a wavelength range of 280–1200 nm was used to investigate the optical transmission spectra of the films. Band gap values were calculated using the equation of Tauc and Menth.

13 $$ahv = A\left( {hv - E_{g} } \right)^{\eta }$$

where *α* is the absorptivity, *h* is Planck's constant, *v* is the frequency of the radiation, *Eg* is the visual indirect band gap, *A* is the constant of proportionality, and *η* is the type of optical transition that occurs as a result of photon absorption.

### Preparation of the photocatalytic process

The photocatalytic performance of the tenorite thin film was evaluated by measuring the degradation of MB under solar irradiation in weather conditions recorded on October 15, 2022, in Biskra, Algeria. A stock solution of 2.2 ppm MB was prepared by dissolving MB in 1 L of double distilled water, and then stirring for 15 min. The solution was then stored in a dark place for 30 min. Each film with an area of approximately 20 cm^2^ was placed in 100 ml of MB solution and positioned at an angle of 37° ± to align with the sun's rays in Biskra. Photocatalytic degradation was monitored for 180 min, with measurements of air temperature, wind speed, and humidity taken at regular intervals. During the reaction, 2.5 ml of the mixture was taken every 45 min and the change in absorbance was measured using a spectrophotometer (UV–VIS JASCO V-770) at a wavelength range of 300–800 nm. To confirm the photocatalytic activity of the tenorite films, the MB solution was exposed under sunlight without the film as well as in a dark place for 3 h. The rate of photocatalytic efficiency (% degradation) was calculated using the following equation:

14 $$\%\,of\,degradation = \left[ {\left( {C_{0} - C_{t} } \right)/C_{0} } \right]*100$$

where *C*_*0*_ and *C*_*t*_ are the dye (MB) concentration (C_0_ is the initial concentration at 0 time and C_t_ at t time). The equation of the constant rate (K_app_) and the correlation coefficient (*R*^2^) was calculated using the following equation:

15 $$r = - dC. dt = K_{app} .C$$

where r is the rate, C is the concentration, t is the time evolution, and K_app_ (min^-1^) is the pseudo-1^st^ rate constant.

## Conclusion

The study investigated the impact of precursor concentrations variation on the properties of tenorite thin films prepared by sol–gel processes and applied to the films by the dip-coating method. The results showed that with the increase of precursor concentrations of tenorite from 0.01 to 0.1 g/ml, the size of the crystal structure increased (from 0.04 to 0.1 g/ml), the particle size distribution became wider (from 13 to 72 nm), the surface of the films became more hydrophobic (from 23.7 to 65.6°), the thickness of the films increased (from 28.6 to 1276.8 nm), and the surface roughness increased (from 1.41 to 77.3 nm). The wettability test showed that the surface of the 0.1 g/ml film improved by 21.47% in indirect sunlight compared to the darkness. Analysis of surface morphology showed that the appearance of grains on the surface of the films increased with increasing precursor concentrations. The transmittance rates of the films at 600 nm ranged from 0.02% to 90.94%. The direct optical band gaps of the films ranged from 2.74 to 1.21 eV, and the indirect band gaps ranged from 1.05 to 1.11 eV, indicating that the indirect band gaps were not affected by precursor concentrations variation. The photocatalytic efficiency of the films toward dyes (MB) was affected by the precursor concentrations of tenorite, crystal phase, crystal size, thickness, wettability, surface roughness, and direct band gap. These tenorite thin films are unique photocatalysts that are highly efficient and environmentally friendly due to their heterogeneous and wettable surfaces, hydroxyl radicals, and superoxide anions. In addition, the study found that these tenorite thin films are highly reusable and stable, making them ideal for real-world applications.

## Data Availability

All data generated or analysed during this study are included in this published article.
